# Influence of cephalomedullary nail length and caput–collum–diaphyseal angle on tip–apex distance and early mechanical cut-out in trochanteric femur fractures

**DOI:** 10.1186/s12891-026-09685-1

**Published:** 2026-03-07

**Authors:** Robert Philipp, Anke Kalb, Dmitry Notov, Christian Kleber, Ulrich Spiegl

**Affiliations:** 1 Department of Orthopaedic and Trauma Surgery, Leipzig University Hospital, Leipzig, Germany; 2 Department of Orthopaedics, Trauma Surgery and Reconstructive Surgery, München Klinik Harlaching, Munich, Germany

**Keywords:** hip fracture, tip–apex distance (TAD), lag screw cut-out, cephalomedullary nail, elderly patients, CCD angle

## Abstract

**Background:**

Early mechanical cut-out remains one of the most severe complications after cephalomedullary fixation of trochanteric femur fractures. While tip–apex distance (TAD) and lag screw position are well-established predictors, the independent influence of nail length and the caput–collum–diaphyseal (CCD) angle remains unclear. This question is clinically relevant because implant standardisation may constrain screw trajectory in anatomically variable proximal femora and thereby hinder optimal TAD attainment and potentially increase cut-out risk.

**Methods:**

We retrospectively analysed 373 consecutive, predominantly older adults with AO/OTA 31-A1 to 31-A3 trochanteric femur fractures treated with cephalomedullary nails at a Level I trauma center (2018–2022). Primary outcomes were achievement of TAD < 25 mm and early mechanical cut-out within 90 days. Multivariable logistic regression and Firth-penalised models addressed confounding and rare-event bias. A predefined homogeneous subgroup of AO/OTA 31-A2 fractures with good reduction (Baumgaertner criteria) treated with 130° nails was analysed separately. Contralateral CCD angle and CCD mismatch were assessed where imaging permitted. Cut-out-free survival was analysed using Kaplan–Meier estimates and penalised Cox regression.

**Results:**

TAD < 25 mm was achieved in 313/373 (83.9%) and center–center screw position in 324/373 (86.9%). Early cut-out occurred in 16/351 (4.6%), with no events after day 65. Nail length and CCD angle were not independently associated with TAD attainment or cut-out (e.g., nail length OR 1.27, 95% CI 0.55–2.91 for TAD < 25 mm; all *p* > 0.30). Center–center positioning strongly predicted TAD < 25 mm (OR 14.98, 95% CI 7.35–30.54; *p* < 0.001) and was associated with lower cut-out risk (OR 0.23, 95% CI 0.09–0.69; *p* = 0.010). TAD < 25 mm was also associated with lower cut-out risk (OR 0.29, *p* = 0.026). CCD mismatch was more pronounced in non-130° configurations but was not associated with early cut-out.

**Conclusion:**

Within the implants used in this cohort, nail length and CCD angle were not independently associated with TAD attainment or early cut-out. Center–center lag screw placement and achieving TAD < 25 mm were the dominant modifiable intraoperative targets to minimise cut-out risk. Although CCD mismatch did not translate into higher cut-out rates in this cohort, it may still be relevant in atypical anatomies and warrants further study.

**Supplementary Information:**

The online version contains supplementary material available at 10.1186/s12891-026-09685-1.

## Introduction

Proximal femur fractures are among the most frequent injuries in elderly patients and account for up to 10% of hospital admissions in individuals over 60 years of age, with incidence rising substantially as populations age [1–3]. Given demographic trends, the incidence of these fractures is expected to increase further, imposing a growing socioeconomic burden on healthcare systems [4]. For many patients, trochanteric femur fractures represent life-altering events associated with prolonged immobility, functional decline, and increased mortality [5, 6].

Trochanteric fractures constitute approximately half of all proximal femoral fractures, and early operative management remains the gold standard to minimise morbidity and mortality [7, 8]. Cephalomedullary nailing has become the preferred fixation method—particularly for unstable fracture patterns—due to superior biomechanical characteristics and lower complication rates compared with extramedullary devices [9, 10]. Despite these advantages, early cut-out remains a relevant complication. In contemporary series of trochanteric fractures treated with cephalomedullary nails, reported cut-out rates are typically in the low single-digit range, although higher rates have been described depending on fracture morphology and technical factors [11, 12]. Cut-out typically results from varus collapse with superior migration of the head element through cancellous bone.

Well-established determinants of cut-out include tip–apex distance (TAD), lag screw positioning within the femoral head, and the quality of fracture reduction. Multiple biomechanical and clinical studies support maintaining a TAD below 25 mm to minimise cut-out risk [13–15]. However, recent work has questioned how universally applicable a single numerical cut-off is, particularly for blade-based head elements and modern implant designs [16–18]. As our cohort comprised lag screw constructs, we retained TAD < 25 mm as a pragmatic benchmark and assessed its performance in relation to early cut-out. In contrast, the influence of implant geometry—particularly femoral nail length and the caput–collum–diaphyseal (CCD) angle—on screw trajectory, the ability to achieve optimal TAD, and the risk of early cut-out remains insufficiently understood. Prior studies report conflicting findings, and many were limited by small sample sizes, heterogeneous fracture patterns, or incomplete radiographic evaluation [13, 14, 19, 20].

A practical and clinically relevant question for trauma surgeons is whether different CCD configurations need to be kept in inventory and selectively implanted based on patient anatomy, or whether a standardised 130° cephalomedullary nail is sufficient for most trochanteric fractures. Examples include pronounced coxa vara (low native CCD) or coxa valga, where trajectory constraints may compromise center–center placement or an optimal TAD. Furthermore, it remains unclear whether CCD mismatch—the angular deviation between the native CCD angle and the implant angle—impairs the ability to achieve center–center screw placement or optimal TAD, potentially increasing cut-out risk.

The present study aimed to address these uncertainties by analysing a large consecutive cohort of patients with AO/OTA 31-A1 to 31-A3 trochanteric fractures treated with short or long cephalomedullary nails. To avoid mixing entities, we focused on trochanteric fractures in the AO/OTA 31-A range and did not include primary subtrochanteric (AO/OTA 32) or diaphyseal femoral fractures. Specifically, we examined three questions: (1) whether nail length or CCD angle affects the likelihood of achieving a TAD < 25 mm or experiencing early cut-out; (2) whether center–center screw positioning is an independent predictor of mechanical stability; and (3) whether CCD mismatch between the native and implanted angles correlates with TAD or early cut-out. To reduce biomechanical heterogeneity and isolate potential effects of nail length, we additionally analysed a predefined homogeneous subgroup consisting of well-reduced AO 31-A2 fractures treated with 130° nails.

Compared with prior work on TAD and cut-out, the present study combines a large consecutive cohort with detailed quantification of contralateral CCD mismatch, a predefined homogeneous AO/OTA 31-A2 subgroup treated with 130° nails to isolate the effect of nail length, and time-to-event analysis focused on early cut-out.

We hypothesised that nail length and CCD angle would show limited independent association with achieving TAD < 25 mm or with early cut-out, whereas center–center screw placement and optimal TAD would remain the strongest modifiable predictors of mechanical success. The primary radiographic endpoint was achievement of TAD < 25 mm on immediate postoperative radiographs, and the key secondary clinical endpoint was early lag screw cut-out within 90 days after surgery.

## Methods

### Study design and patient selection

**Fig. 1:**
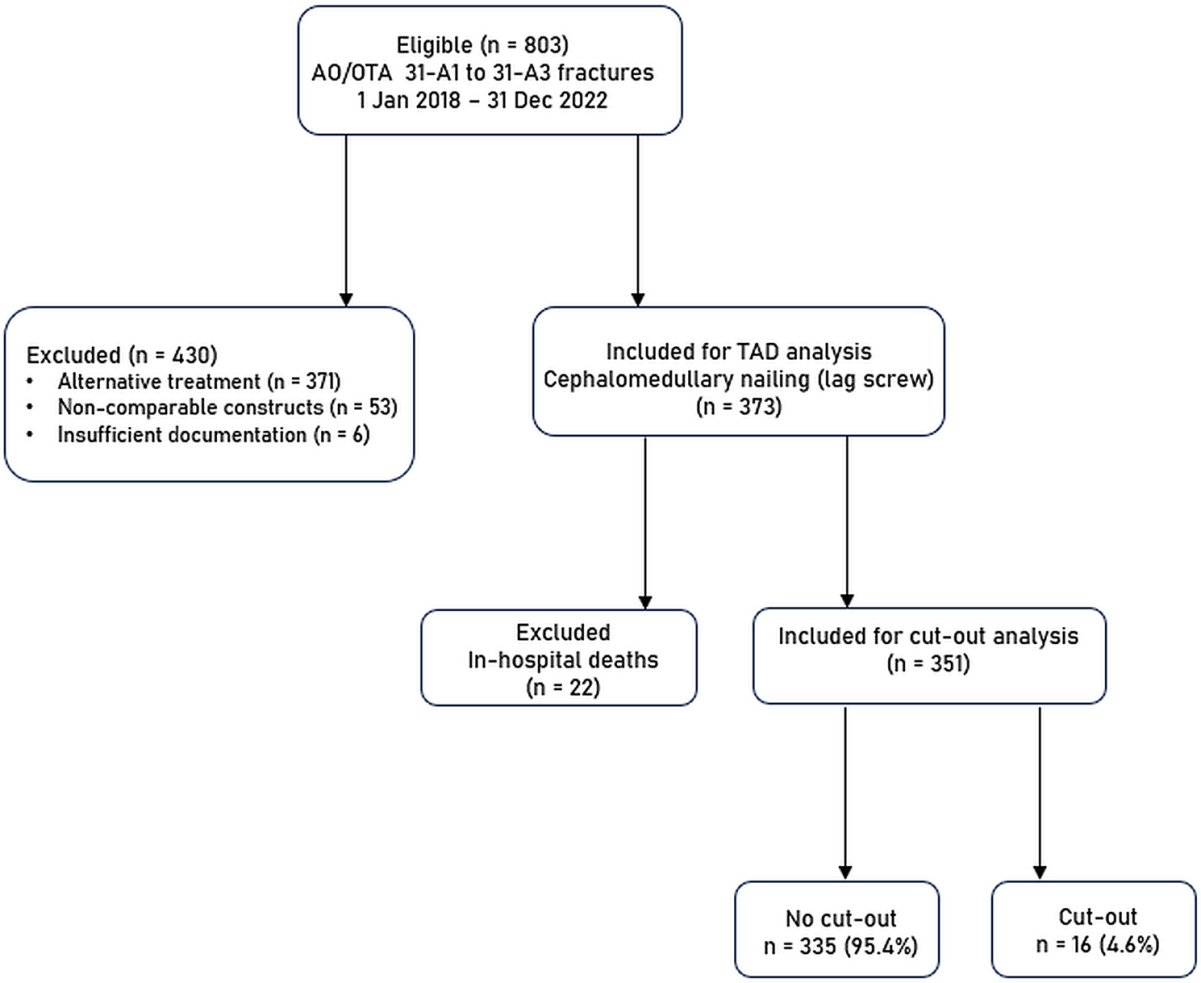
Flowchart of patient selection and analysis inclusion. Overview of patient eligibility, exclusions, and final inclusion in the TAD and cut-out analyses

This retrospective cohort study included all consecutive patients treated for trochanteric femoral fractures (AO/OTA 31-A1 to 31-A3) at a supra-regional level I trauma center between January 1, 2018, and December 31, 2022 (Fig. 1). Eligible cases were identified through the institutional discharge database (SAP). Overall, 803 patients were screened (Fig. 1). Patients were included if they were treated with an intramedullary cephalomedullary femoral nail system and if complete clinical, operative, and radiographic documentation was available in the electronic records and PACS (Sectra/HYDMedia). Only one fracture episode per patient was included to ensure independence of observations. If a patient sustained bilateral fractures during the study period, only the first eligible fracture episode was included.

Exclusion criteria were grouped into three categories (Fig. 1). Alternative treatment comprised cases not treated with standard cephalomedullary lag screw nailing as the index procedure, including extramedullary fixation (e.g., DHS/plate constructs), arthroplasty procedures, pathological fractures, revision procedures, and patients with relevant additional ipsilateral lower-limb injuries. Non-comparable constructs comprised cephalomedullary constructs not comparable to standard lag screw nailing due to modified or augmented fixation, including supplementary cerclage and/or plate fixation (*n* = 29), blade-based head elements (*n* = 12), cement augmentation (*n* = 8), and external fixation (*n* = 4). Insufficient documentation comprised missing operative documentation and/or inadequate postoperative radiographs preventing reliable measurements. Patients who died during their initial hospital stay were excluded from cut-out analyses to reduce competing-risk distortion (*n* = 22; Fig. 1) but were retained in the radiographic TAD analyses.

Postoperative events and complications were identified by systematic review of the institutional electronic medical record. Data collection was initiated only after at least six months had elapsed from the index operation of the last included patient (December 2022), thereby ensuring a minimum potential follow-up of six months for every case within the hospital information system. For each patient, all available inpatient notes, outpatient clinic reports, emergency department contacts and radiology reports were reviewed.

For the analysis of early mechanical cut-out, a 90-day observation window was defined a priori to focus on early postoperative failures, as lag screw cut-out is generally regarded as an early complication. Within this period, any documentation of new hip pain, loss of function, or radiographic reassessment of the operated hip was screened for evidence of cut-out, defined as radiographic migration/penetration of the lag screw through the femoral head. The primary radiographic outcome was achievement of TAD < 25 mm on immediate postoperative radiographs, and the key secondary clinical outcome was lag screw cut-out.

### Data collection

All data were abstracted using study-specific identifiers and pseudonymised. Baseline variables included age, sex, body mass index, ASA classification, AO/OTA fracture type, trauma mechanism (low- vs. high-energy), and comorbidities classified into 17 predefined categories (Supplementary Table S1). These categories reflect an institutional, pragmatic grouping for chart abstraction and were not derived from a validated summary index such as the Charlson Comorbidity Index. Medication status at admission was grouped as none, oral anticoagulation, osteoporosis treatment, or both and was not captured at the level of specific agents, dosages, or treatment duration (Supplementary Table S2). Information on osteoporosis management at discharge was not available as a structured variable beyond medication status at admission.

Operative data included surgical side, duration, surgeon experience (categorised as senior staff present vs. not), hospital length of stay, nail length (short ≤ 245 mm vs. long > 245 mm), nail diameter, CCD angle (125°, 130°, or 135°; 125° and 135° were pooled as non-130° when applicable), lag screw length and diameter, reduction method (closed or open), and intraoperative complications. When specified in the operative report, complications were categorised as technical/surgical problems, instrumentation/implantation problems, haemodynamic instability, coagulation-related events, operative delay, or other (Supplementary Table S3). Distal locking configuration was not captured as a structured variable and could therefore not be analysed. Likewise, postoperative weight-bearing instructions, the presence of formal geriatric co-management, and infectious complications were not recorded in a standardised manner. The cephalomedullary nail systems used were Zimmer CMN (Zimmer Biomet, Warsaw, IN, USA) and TFN-Advanced (TFNA) (DePuy Synthes, Raynham, MA, USA). The included cohort underwent cephalomedullary nailing with a single proximal lag screw. The indication for selecting a long versus short nail was not standardised in the database and was not captured as a structured variable; implant choice followed routine surgeon decision-making and institutional availability. During the study period, long cephalomedullary nails were routinely available only in a 130° CCD configuration, whereas short nails were available in 125°, 130°, and 135° CCD configurations.

Radiographic variables comprised screw position, TAD, and fracture reduction quality. Postoperative medical complications were captured as predefined in-hospital events (Supplementary Table S4), and the total number of postoperative medical complications per patient was recorded (Table 1).

**Table 1:** Baseline characteristics of the full cohort (*n* = 373)

Variable	Category	Total (*n* = 373)	Short nails (*n* = 249)	Long nails (*n* = 124)	*p*-value
Gender (n, %)	Male	139 (37.3)	96 (38.6)	43 (34.7)	0.538
	Female	234 (62.7)	153 (61.4)	81 (65.3)	
Time of operationᵃ (n, %)	Morning	106 (28.4)	71 (28.5)	35 (28.2)	0.337
	Afternoon	140 (37.5)	99 (39.8)	41 (33.1)	
	Evening/Night	127 (34.1)	79 (31.7)	48 (38.7)	
Surgical team experienceᵇ (n, %)	​​≥ 1 senior surgeon​	313 (83.9)	202 (81.1)	111 (89.5)	0.054
	No senior surgeon	60 (16.1)	47 (18.9)	13 (10.5)	
Operative side (n, %)	Right	196 (52.6)	139 (55.8)	57 (46.0)	0.092
	Left	177 (47.4)	110 (44.2)	67 (54.0)	
Nail type (n, %)	Zimmer CMN (Zimmer Biomet)	353 (94.6)	232 (93.2)	121 (97.6)	0.089
	TFNA (DePuy Synthes)	20 (5.4)	17 (6.8)	3 (2.4)	
Implant CCD angle (n, %)	125°	12 (3.2)	12 (4.8)	0 (0.0)	< 0.001
	130°	293 (78.6)	169 (67.9)	124 (100.0)	
	135°	68 (18.2)	68 (27.3)	0 (0.0)	
Reduction method (n, %)	Open	92 (24.7)	34 (13.7)	58 (46.8)	< 0.001
	Closed	281 (75.3)	215 (86.3)	66 (53.2)	
Intraoperative complications (n, %)	None	315 (84.5)	221 (88.8)	94 (75.8)	0.002
	Yes	58 (15.5)	28 (11.2)	30 (24.2)	
Mechanism of injury (n, %)	Low-energy	356 (95.4)	245 (98.4)	111 (89.5)	< 0.001
	High-energy	17 (4.6)	4 (1.6)	13 (10.5)	
Osteoporosis treatment (n, %)	None	262 (70.2)	171 (68.7)	91 (73.4)	0.414
	Yes	111 (29.8)	78 (31.3)	33 (26.6)	
ASA classification (n, %)	I	8 (2.1)	4 (1.6)	4 (3.2)	0.062
	II	104 (27.9)	60 (24.1)	44 (35.5)	
	III	243 (65.2)	173 (69.5)	70 (56.5)	
	IV	18 (4.8)	12 (4.8)	6 (4.8)	
AO classification (n, %)	31-A1	20 (5.4)	19 (7.6)	1 (0.8)	< 0.001
	31-A2	254 (68.1)	211 (84.7)	43 (34.7)	
	31-A3	99 (26.5)	19 (7.6)	80 (64.5)	
Cut-out (≤ 90 days)ᶜ (n, %)	No	335 (95.4)	224 (95.0)	111 (96.5)	0.594
	Yes	16 (4.6)	12 (5.0)	4 (3.5)	
Reduction qualityᵈ (n, %)	Good	273 (73.2)	206 (82.7)	67 (54.0)	< 0.001
	Acceptable	94 (25.2)	40 (16.1)	54 (43.5)	
	Poor	6 (1.6)	3 (1.2)	3 (2.4)	
Screw positionᵉ (n, %)	Center–center	324 (86.9)	217 (87.1)	107 (86.3)	0.152
	Other	49 (13.1)	32 (12.9)	17 (13.7)	
TAD < 25 mmᶠ (n, %)	No	60 (16.1)	40 (16.1)	20 (16.1)	1.000
	Yes	313 (83.9)	209 (83.9)	104 (83.9)	
Patient age median (IQR, Q1 to Q3) (years)		83 (75–89)	84 (77–89)	80 (69–88)	0.003
Length of hospital stay median (IQR, Q1 to Q3) (days)		9 (6–13)	9 (6–13)	9 (7–13)	0.569
Operative duration median (IQR, Q1 to Q3) (min)		70 (51–102)	59 (46–81)	100 (77–133)	< 0.001
BMI median (IQR, Q1 to Q3) (kg/m^2^)		25 (22–28)	25 (21–27)	24.5 (23–28)	0.060
Number of comorbidities median (IQR, Q1 to Q3)		4 (2–5)	4 (3–5)	3 (2–5)	0.027
Number of postoperative medical complications median (IQR, Q1 to Q3)		0 (0–1)	0 (0–1)	0 (0–1)	0.504
TAD medianᶠ (IQR, Q1 to Q3) (mm)		19.2 (15.4–23.2)	19.4 (15.4–23.3)	18.4 (15.5–23.1)	0.818

*BMI *body-mass index, *ASA *American Society of Anesthesiologists physical status, *AO *AO Foundation fracture classification, *CCD *caput–collum–diaphyseal angle, *TAD *tip–apex distance, *IQR *interquartile range (Q1–Q3)

ᵃ Time categories: Morning = 08:00–11:59; Afternoon = 12:00–16:59; Evening/Night = 17:00–07:59

ᵇ Team experience: ≥1 senior surgeon vs. none

ᶜ Cut-out analysis performed in *n* = 351 after excluding in-hospital deaths (*n* = 22)

ᵈ Baumgaertner criteria [14]

ᵉ Modified Cleveland zones [21]

ᶠ TAD = tip–apex distance, corrected for magnification [14]

### Radiographic Assessment

All radiographic parameters were primarily assessed by the first author. To ensure accuracy and consistency, all measurements (including TAD and CCD angles) were subsequently reviewed and verified by a senior trauma surgeon. In cases of disagreement, a consensus was reached. Radiographic parameters were systematically assessed on standardised postoperative radiographs acquired immediately after surgery using Sectra PACS software. Imaging protocols included an anteroposterior (AP) view with approximately 15° of internal limb rotation and a cross-table lateral view with the contralateral leg flexed and abducted.

#### Tip–Apex Distance (TAD)

**Fig. 2:**
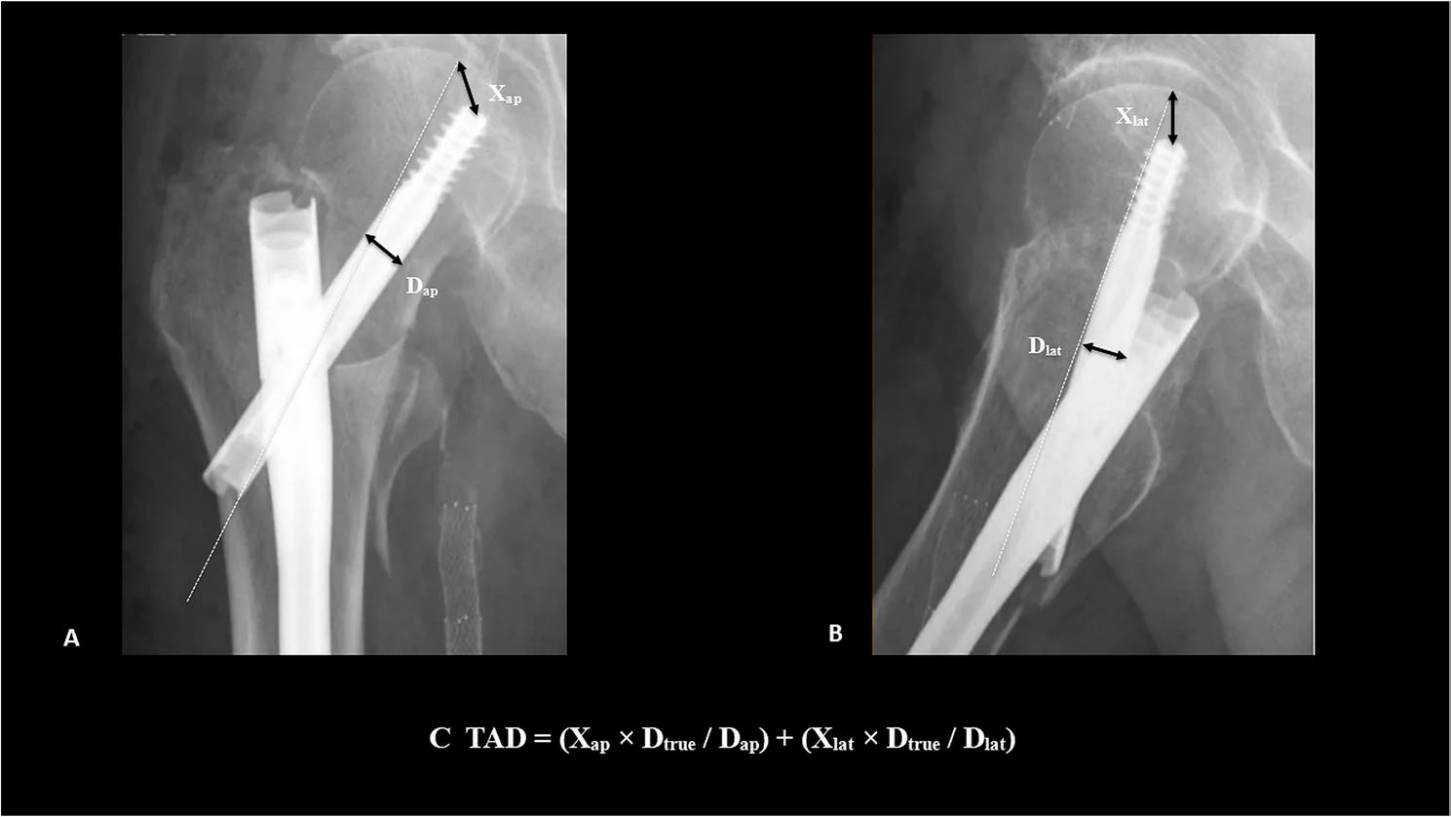
Radiographic measurement of tip–apex distance (TAD). Postoperative anteroposterior (A) and lateral (B) radiographs illustrating the measurement of the TAD according to the method described by Baumgaertner et al. [14]. X represents the distance from the screw tip to the femoral head apex; D denotes the measured screw diameter on each projection; Dₜ_r__u_ₑ is the true implant diameter. (C) shows the formula used to calculate the total TAD, incorporating both AP and lateral projections with magnification correction. Dashed lines represent the femoral neck axis. Measurement arrows and distances were added for illustration

TAD was calculated using Baumgaertner’s validated method [14]. The femoral head apex was defined as the intersection of the subchondral bone and a line drawn parallel to and through the center of the femoral neck. The sum of distances from the tip of the lag screw to the apex, measured on AP and lateral radiographs, yielded the final TAD value, corrected for radiographic magnification (Fig. 2).

#### Lag screw positioning

**Fig. 3:**
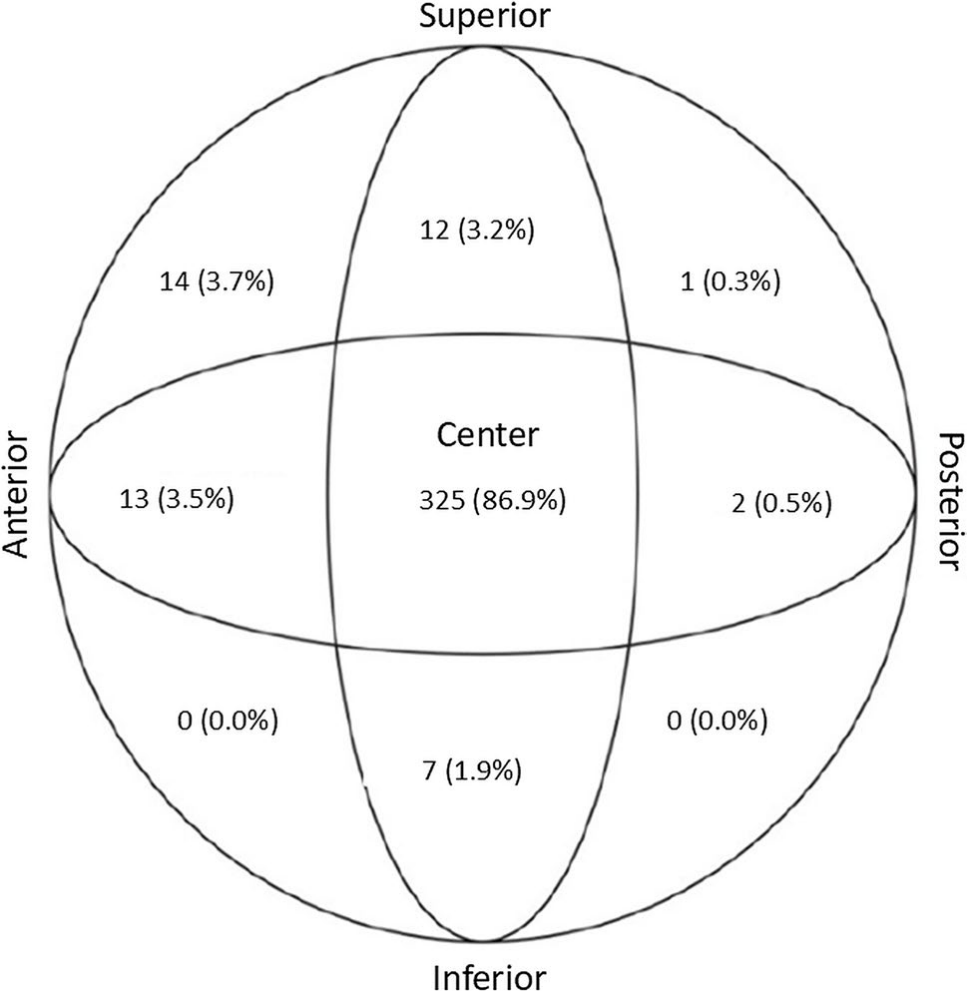
Screw positioning within the femoral head according to modified Cleveland zones. Distribution of lag screw positions (*n* = 373) according to the modified Cleveland zone classification [21]. The first number in each zone indicates the number of lag screws placed in that position, and the value in parentheses indicates the percentage of all screw placements

Screw position within the femoral head was assessed using a modified Cleveland zone classification [21]. Both AP and lateral views were divided into three equal zones—superior, center, and inferior (AP), and anterior, center, and posterior (lateral)—yielding nine distinct sectors for accurate documentation of screw placement (Fig. 3). Screw position was recorded across all nine modified Cleveland zones. For the primary analyses, positions were dichotomised a priori as center–center versus non-center.

#### Reduction quality

Fracture reduction was evaluated according to Baumgaertner’s criteria on immediate postoperative radiographs [14]. Alignment was considered optimal if anatomical or slightly valgus (< 10° deviation on AP, < 20° on lateral view). Displacement was deemed acceptable if fracture gaps were ≤ 4 mm. Reduction quality was categorised as good (both criteria met), acceptable (one criterion met), or poor (neither criterion met).

#### Contralateral CCD angle and mismatch.

Because fracture displacement, rotational malalignment, and varus collapse render CCD measurements of the fractured side unreliable, the native CCD angle was measured on the contralateral hip, which exhibits high anatomical symmetry in adults [19, 22]. CCD mismatch was defined as the absolute angular difference between the native CCD angle and the implant angle. Patients with contralateral implants or inadequate imaging were excluded from this analysis; consequently, contralateral CCD measurements were available in 341/373 patients (32 excluded).

### Statistical analysis

All analyses were performed using R (version 4.3.2, R Foundation for Statistical Computing, Vienna, Austria). A two-sided p-value < 0.05 was considered statistically significant. Categorical variables are presented as frequencies and percentages, and continuous variables as medians with interquartile ranges (IQR) after assessment of normality using the Shapiro–Wilk test. Group comparisons were conducted using χ² or Fisher’s exact tests for categorical variables and Student’s t-test or Mann–Whitney U test for continuous variables, as appropriate.

To ensure transparency and facilitate interpretation across multiple related analyses, statistical modelling followed a predefined stepwise analytical strategy aligned with the study objectives:

Descriptive comparisons. Baseline, fracture-related, and operative characteristics were compared between short- and long-nail constructs to describe differences in case mix.

Full cohort—predictors of TAD attainment. In the full cohort, multivariable logistic regression was used to assess whether nail length (short vs. long) was independently associated with achieving TAD < 25 mm, adjusting for screw position, age (per 1-year increase), fracture classification, and reduction quality. Linearity of continuous predictors in the logit was assessed using the Box–Tidwell test; no relevant non-linearity was detected. Multicollinearity was evaluated using variance inflation factors (VIF), with all VIF values < 2.

Predefined homogeneous subgroup. To reduce biomechanical heterogeneity and isolate the effect of nail length under comparable implant geometry, a predefined subgroup of well-reduced AO/OTA 31-A2 fractures (Baumgaertner criteria) treated with 130° nails was analysed separately. Cases with acceptable or poor reduction were retained in the full-cohort and short-nail analyses and adjusted for in multivariable models; they were excluded only from this predefined subgroup. Because event counts were low, Firth-penalised logistic regression was applied for both TAD < 25 mm and cut-out endpoints to mitigate small-sample bias (logistf package, R).

Short-nail subgroup—CCD configuration and TAD. As different CCD configurations were only available in short nails, analyses evaluating implant CCD angle (130° vs. non-130°) were restricted to the short-nail subgroup using multivariable logistic regression with TAD < 25 mm as the dependent variable (adjusting for screw position and reduction quality).

Predictors of early mechanical cut-out. Because cut-out events were uncommon, Firth-penalised logistic regression was used to estimate odds ratios for cut-out within 90 days. Patients who died during the index hospital stay were excluded from cut-out analyses to reduce distortion by competing risk.

Contralateral CCD mismatch analyses. CCD mismatch was defined as the absolute difference between the native contralateral CCD angle and the implanted CCD configuration. Group differences were assessed using parametric or non-parametric tests as appropriate, and exploratory associations with TAD attainment and cut-out were evaluated in clinically relevant strata (e.g. well-reduced short-nail constructs).

Time-to-event analysis. Kaplan–Meier methods and Cox proportional hazards models were used to evaluate cut-out-free survival within 90 days, with censoring at 90 days. The proportional hazards assumption was assessed using Schoenfeld residuals and was met for all models. Given sparse events, penalised Cox regression was applied to reduce small-sample bias (coxphf package, R).

Results are reported as odds ratios (OR) or hazard ratios (HR) with 95% confidence intervals (CI). Model robustness was explored using bootstrap resampling (1,000 iterations). All figures were generated in R and finalised using Microsoft PowerPoint.

### Ethical considerations

The study adhered to the Declaration of Helsinki and was approved by the Institutional Research Ethics Committee (Approval No.: 166‑20‑ek). Owing to its retrospective design and pseudonymised data collection, informed consent was waived. The manuscript was prepared in accordance with the STROBE guidelines [22]. Data were pseudonymised and handled in accordance with institutional data protection regulations.

## Results

### Study population and baseline characteristics

Among 803 screened patients (2018–2022), 373 met inclusion criteria for the radiographic tip–apex distance (TAD) analysis. For cut-out analyses, 351 patients were available after excluding 22 in-hospital deaths (5.9%). The cohort had a median age of 83 years (IQR 75–89), and 62.7% were female. Most fractures were AO/OTA 31-A2 (68.1%), followed by 31-A3 (26.5%) and 31-A1 (5.4%). Low-energy trauma accounted for 95.4% of cases, and 65.2% were ASA class III. Median operative duration was 70 min (IQR 51–102), and median hospital stay was 9 days (IQR 6–13). Closed reduction was performed in 75.3% of cases, and reduction quality was graded as good in 73.2%, acceptable in 25.2%, and poor in 1.6% (Table 1). Lag screw position was center–center in 86.9%. Within 90 days, cut-out occurred in 16/351 patients (4.6%), and TAD < 25 mm was achieved in 313/373 patients (83.9%) (Table 1). Patients who died during the index hospital stay were older (median 89 [IQR 83–92] vs. 82 [IQR 74–88] years; *p* < 0.001), and ASA class distribution was shifted towards higher classes compared with patients discharged alive (*p* < 0.001).

### Descriptive comparison between short and long nails

Patients treated with long nails (*n* = 124) were younger (median 80 vs. 84 years; *p* = 0.003), had longer operative duration (median 100 vs. 59 min; *p* < 0.001), and more intraoperative complications (24.2% vs. 11.2%; *p* = 0.002). Long nails were more frequently used in AO 31-A3 fractures (64.5% vs. 7.6%; *p* < 0.001), and open reductions were more common (46.8% vs. 13.7%; *p* < 0.001). The distribution of intraoperative complication subtypes is provided in Supplementary Table S3. The median number of comorbidity categories was 4 (IQR 3–5) in short nails and 3 (IQR 2–5) in long nails (*p* = 0.027) (Table 1; Supplementary Table S1). Cut-out rates (≤ 90 days; excluding in-hospital deaths) were 12/240 (5.0%) in short nails and 4/111 (3.5%) in long nails (*p* = 0.594). The proportion achieving TAD < 25 mm did not differ between short and long nails (209/249 [83.9%] vs. 104/124 [83.9%]; *p* = 1.000) (Table 1).

### Full cohort—predictors of TAD < 25 mm

In multivariable logistic regression, nail length was not associated with achieving TAD < 25 mm (OR 1.27; 95% CI 0.55–2.91; *p* = 0.575). Center–center screw positioning was the strongest predictor (OR 14.98; 95% CI 7.35–30.54; *p* < 0.001). Age was associated with higher odds of achieving TAD < 25 mm (OR 1.03 per year; 95% CI 1.01–1.05; *p* = 0.011). Fracture classification (OR 0.90; 95% CI 0.36–2.22; *p* = 0.813) and reduction quality (poor vs. good: OR 0.69; 95% CI 0.08–6.20; *p* = 0.737) were not significant in the adjusted model (Table 2; Supplementary Fig. S1).

**Table 2:** Multivariable logistic regression predicting TAD < 25 mm (*n* = 373)

Variable	OR (95% CI)	*p*-value
Nail lengthᵃ	1.27 (0.55–2.91)	0.575
Screw positionᵇ	14.98 (7.35–30.54)	< 0.001
Age (per year)	1.03 (1.01–1.05)	0.011
Fracture classificationᶜ	0.90 (0.36–2.22)	0.813
Reduction qualityᵈ	0.69 (0.08–6.20)	0.737

OR = odds ratio; CI = confidence interval

ᵃ Short vs. long nail

ᵇ Center vs. non-center screw position by modified Cleveland zones [21]

ᶜ AO/OTA A3 vs. A1/A2 fracture classification

ᵈ Poor vs. good reduction according to Baumgaertner et al. [14]

### Predefined homogeneous subgroup—well-reduced AO 31-A2 fractures treated with 130° nails

In the predefined subgroup of well-reduced AO 31-A2 fractures treated with 130° nails, 148 patients were available for the TAD analysis. For cut-out analyses, 139 patients were available after excluding in-hospital deaths; 6 cut-out events occurred in this subgroup. In univariate Firth regression, nail length was not associated with achieving TAD < 25 mm (OR 0.86; 95% CI 0.28–3.21; *p* = 0.799) or with cut-out (OR 1.22; 95% CI 0.12–6.50; *p* = 0.837). Corresponding 95% confidence intervals were wide.

### Short-nail subgroup—CCD configuration and TAD < 25 mm

Among patients treated with short nails (*n* = 249), CCD configuration (130° vs. non-130°) was not associated with achieving TAD < 25 mm (OR 1.29; 95% CI 0.57–2.93; *p* = 0.540). Center–center screw positioning remained strongly associated with TAD < 25 mm (OR 16.78; 95% CI 7.09–39.71; *p* < 0.001), whereas reduction quality (poor vs. good) was not significant (OR 0.62; 95% CI 0.03–14.12; *p* = 0.767) (Table 3; Supplementary Fig. S2).

**Table 3:** Multivariable logistic regression predicting TAD < 25 mm in short nails (*n* = 249)

Variable	OR (95% CI)	*p*-value
CCD angleᵃ	1.29 (0.57–2.93)	0.540
Screw positionᵇ	16.78 (7.09–39.71)	< 0.001
Reduction qualityᶜ	0.62 (0.03–14.12)	0.767

OR = odds ratio; CI = confidence interval

ᵃ 130° vs. non-130° implant CCD angle

ᵇ Center vs. non-center screw position by modified Cleveland zones [21]

ᶜ Poor vs. good reduction according to Baumgaertner et al. [14]

In exploratory analyses within short nails, nail diameter was not associated with achieving TAD < 25 mm (*p* = 0.596) and was not associated with cut-out (*p* = 0.355). Cut-out occurred in 2/18 (11.1%) for 10-mm nails, 4/87 (4.6%) for 10.5-mm nails, and 6/93 (6.5%) for 11-mm nails; other diameter categories had ≤ 28 cases each.

### Predictors of early mechanical cut-out (≤ 90 days)

Across the full cohort, TAD < 25 mm was associated with lower odds of cut-out (OR 0.29; 95% CI 0.11–0.86; *p* = 0.026) (Table 4; Supplementary Fig. S3). Center–center screw positioning was associated with lower odds of cut-out (OR 0.23; 95% CI 0.09–0.69; *p* = 0.010), and this association was also observed in short nails (OR 0.18; 95% CI 0.06–0.62; *p* = 0.008). Absolute cut-out event counts were 10/303 (3.3%) for center–center screws and 6/48 (12.5%) for non-center screws (*p* = 0.014). Nail length was not associated with cut-out in the full cohort (OR 0.60; 95% CI 0.16–1.87; *p* = 0.470) (Table 4). CCD configuration (130° vs. non-130°) was not associated with cut-out in the short-nail cohort (OR 0.75; 95% CI 0.23–2.57; *p* = 0.630) (Table 4). Due to the low event count, 95% confidence intervals for nail length and CCD configuration were wide and compatible with modest benefit or harm.

**Table 4:** Firth-penalised logistic regression: predictors of implant cut-out (≤ 90 days)

Variable	OR (95% CI)	*p*-value
TAD (all nails)ᵃ	0.29 (0.11–0.86)	0.026
TAD (short nails)	0.34 (0.11–1.24)	0.098
Screw position (all nails)ᵇ	0.23 (0.09–0.69)	0.010
Screw position (short nails)	0.18 (0.06–0.62)	0.008
Nail lengthᶜ	0.60 (0.16–1.87)	0.470
CCD angleᵈ	0.75 (0.23–2.57)	0.630

OR = odds ratio; CI = confidence interval

ᵃ Tip–apex distance < 25 mm vs. ≥ 25 mm [14]

ᵇ Center vs. non-center lag screw position by modified Cleveland zones [21]

ᶜ Short vs. long nail

ᵈ 130° vs. non-130° implant CCD angle within the short-nail cohort (as long nails were available only in 130°)

### Contralateral CCD mismatch analysis

Contralateral CCD measurements were available in 341 patients. Mean CCD mismatch was 4.49° ± 3.58 in 130° implants (*n* = 268) and 5.64° ± 4.38 in non-130° implants (*n* = 73) (*p* = 0.096). In the short-nail subgroup with contralateral measurements (*n* = 222), mismatch differed between 130° and non-130° implants (3.98° ± 3.04 [*n* = 149] vs. 5.64° ± 4.38 [*n* = 73]; *p* = 0.025). In well-reduced short nails, mismatch was 4.02° ± 3.04 (130°) versus 6.08° ± 4.35 (non-130°) (*p* = 0.006). In this subgroup, TAD < 25 mm occurred in 132/149 (88.6%) for 130° versus 57/73 (78.1%) for non-130° implants (*p* = 0.026), while cut-out (2.8% vs. 7.4%; *p* = 0.153) and center–center screw position (88.6% vs. 80.8%; *p* = 0.173) did not differ significantly. In the short-nail contralateral-measurement subset, CCD mismatch was not correlated with operative duration (Spearman ρ = −0.050; *p* = 0.455) and did not differ between open and closed reductions (*p* = 0.374).

### Time-to-event analysis

**Fig. 4:**
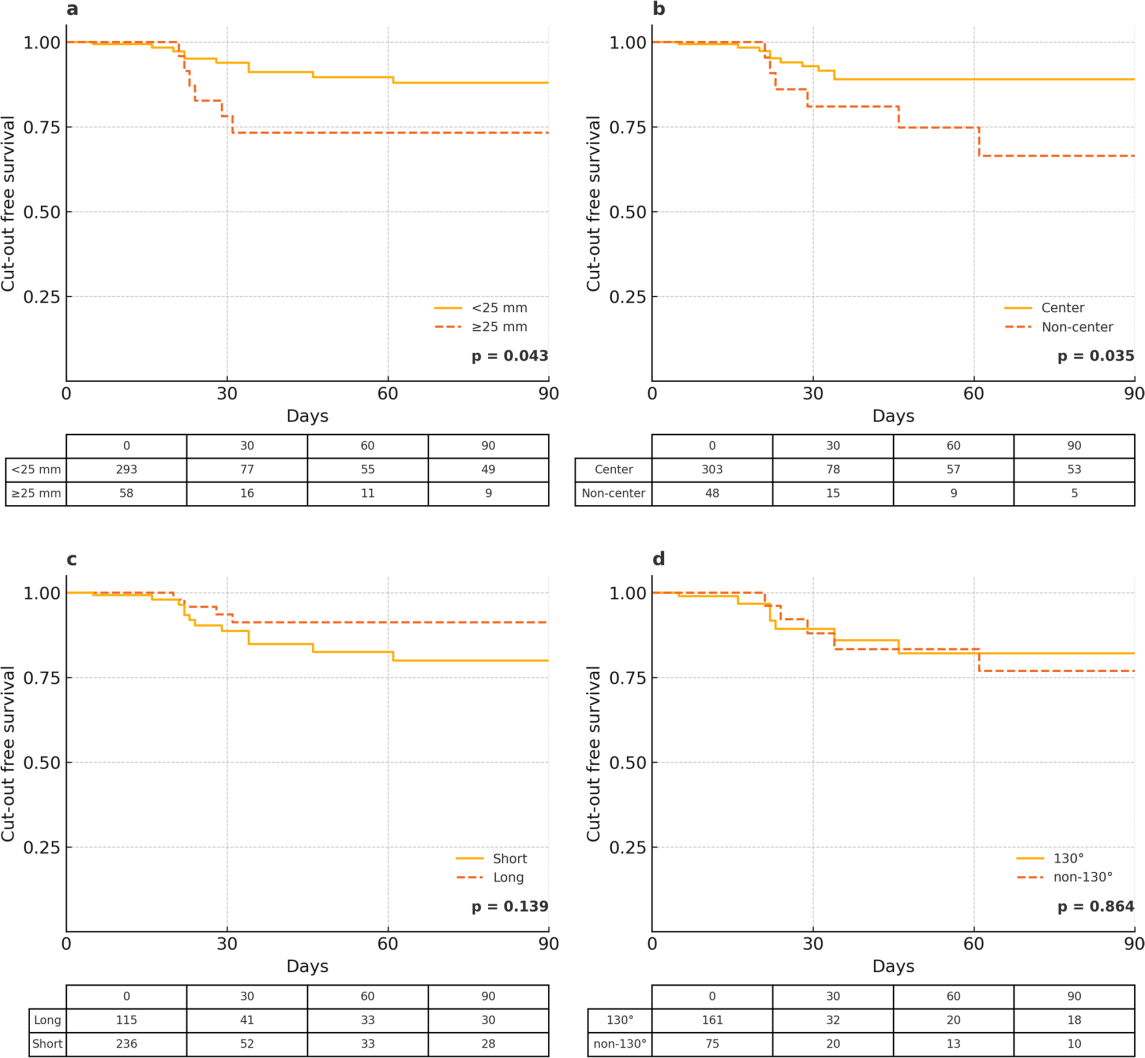
Kaplan–Meier analysis of cut-out-free survival within 90 days. Kaplan–Meier survival curves for four potential predictors of implant cut-out within 90 days postoperatively: (a) TAD, (b) screw position, (c) nail length, and (d) CCD angle in short nails. Cut-out-free survival probabilities are shown over time, along with the number at risk at each interval. Log-rank p-values are presented in each panel. ¹ TAD: < 25 mm vs. ≥ 25 mm; ² Screw position: center vs. non-center, based on modified Cleveland zones [21]; ³ Nail length: short vs. long cephalomedullary nail; ⁴ CCD angle: 130° vs. non-130° implant CCD angle (short nails only). Log-rank significance: (a) *p* = 0.043, (b) *p* = 0.035, (c) *p* = 0.139, (d) *p* = 0.864. Hazard ratios and confidence intervals are reported in Table 5. Note: All cut-out events occurred by postoperative day 65; the x-axis is displayed to 90 days to reflect the predefined censoring window

Kaplan–Meier analysis showed longer cut-out-free survival for patients with TAD < 25 mm (HR 0.36; 95% CI 0.14–0.98; *p* = 0.043) and for center–center screw positioning (HR 0.35; 95% CI 0.13–0.98; *p* = 0.035) (Table 5; Fig. 4). No significant effects were observed for nail length (HR 0.44; 95% CI 0.14–1.35; *p* = 0.139) or CCD configuration (HR 0.63; 95% CI 0.22–1.82; *p* = 0.864). No cut-out events occurred after postoperative day 65.

**Table 5:** Cox proportional hazards regression: hazard ratios for cut-out–free survival within 90 days

Variable	HR (95% CI)	*p*-value
TADᵃ	0.36 (0.14–0.98)	0.043
Nail lengthᵇ	0.44 (0.14–1.35)	0.139
CCD angleᶜ	0.63 (0.22–1.82)	0.864
Screw positionᵈ	0.35 (0.13–0.98)	0.035

HR = hazard ratio; CI = confidence interval

ᵃ Tip–apex distance < 25 mm vs. ≥ 25 mm [14]

ᵇ Short vs. long nail

ᶜ 130° vs. non-130° implant CCD angle within the short-nail cohort (as long nails were available only in 130°)

ᵈ Center vs. non-center lag screw position by modified Cleveland zones [21]

## Discussion

Implant cut-out remains one of the most serious complications following cephalomedullary nailing of proximal femoral fractures, as it substantially compromises mobility and increases mortality—especially in elderly patients [5, 6, 23]. Identifying reliable, modifiable intraoperative parameters to minimise cut-out risk is therefore a clinical priority. The present study evaluated, in a large consecutive cohort, whether femoral nail length and implant caput–collum–diaphyseal (CCD) angle influence the achievement of a tip–apex distance (TAD) < 25 mm and the risk of early implant cut-out, and how these factors compare with the effects of lag screw positioning.

From a clinical standpoint, the relevance of this question lies in everyday decision-making: surgeons and hospitals must decide whether multiple nail designs and CCD configurations need to be kept in stock and selectively implanted based on individual anatomy, or whether a standardised 130° cephalomedullary nail can be used safely in most trochanteric fractures, provided that reduction quality and screw placement are optimised. Our main finding was that implant geometry (nail length and CCD angle) showed no independent association with either achieving TAD < 25 mm or early cut-out, whereas center–center screw positioning and low TAD were strongly and consistently protective. Within the range of currently used implants, technical execution of reduction and screw placement therefore appears to have a much greater impact on mechanical success than the choice of nail length or CCD angle.

### Tip–apex distance as a modifiable target

Since its introduction by Baumgaertner et al. in 1995 [14], tip–apex distance (TAD) has become a widely accepted radiographic predictor of lag screw cut-out, with a threshold of < 25 mm frequently cited to reduce cut-out risk [13–15]. More recent studies have challenged the universality of this cut-off, proposing higher thresholds or questioning the validity of any strict numerical limit [16, 17]. In our study, patients with TAD < 25 mm had a significantly reduced risk of cut-out (OR = 0.29, *p* = 0.026), and Kaplan–Meier analysis showed prolonged cut-out-free survival (HR = 0.36, 95% CI 0.14–0.98; *p* = 0.043). Notably, 16.1% of our cohort had a TAD ≥ 25 mm (60/373), and this proportion was identical in short and long nails (83.9% vs. 83.9% achieved TAD < 25 mm). This relatively high share of “suboptimal” TAD values likely increased statistical contrast between groups and may have strengthened the observed association between TAD and early cut-out in our dataset. In settings where the vast majority of cases consistently meet the TAD target, other determinants of cut-out—such as varus malreduction, lateral wall incompetence, fracture extension patterns, or construct-specific factors—may become more prominent [24]. Importantly, reduction quality was incorporated into our analyses, which may have reduced the apparent independent contribution of several of these emerging risk factors. We therefore interpret TAD < 25 mm as a pragmatic intraoperative benchmark that remains clinically useful, while recognizing that its predictive value depends on the prevalence of TAD ≥ 25 mm in a given cohort. At the same time, our data underscore that TAD should not be interpreted in isolation. TAD is a composite parameter that reflects both screw depth and trajectory; its optimisation depends directly on the ability to achieve a central position within the femoral head. In this sense, TAD represents one important component of a broader biomechanical construct that also includes reduction quality and screw positioning.

### Screw positioning as the key intraoperative determinant

Among all evaluated variables, center–center lag screw positioning was the most robust predictor of favourable outcomes. Center–center positioning increased the odds of achieving TAD < 25 mm by more than an order of magnitude (OR = 14.98; *p* < 0.001), significantly reduced cut-out risk (OR = 0.23; *p* = 0.010), and was associated with longer cut-out-free survival (HR = 0.35; *p* = 0.035). These effects were consistent across short and long nails and in subgroup analyses.

These findings are in line with previous reports emphasising the importance of screw trajectory and central placement within the femoral head [21, 25, 26]. Taken together, they suggest that lag screw trajectory—rather than implant geometry—is the most actionable intraoperative determinant of mechanical success. Clinically, this implies that achieving precise fracture reduction and a true center–center screw position should be prioritised over deliberations about nail length or CCD angle. Pragmatically, achieving a true center–center trajectory depends on projectional accuracy. To translate this into clinical practice, surgeons should prioritise two key maneuvers: first, insisting on a true AP and lateral view of the proximal femur before definitive screw seating to ensure that the projected trajectory matches the 3D anatomy. Second, careful clinical and radiographic rotational alignment of the femur must be verified before final imaging and distal locking to prevent both projectional errors and functional malrotation. Even if the nail’s CCD angle does not perfectly match the patient’s anatomy, these meticulous intraoperative adjustments allow for compensatory positioning of the nail to achieve the targeted center–center placement. Standardising a limited set of implant configurations while focusing training and quality control on reduction and screw placement may therefore be an effective strategy to reduce cut-out rates in high-volume centers.

### Nail length and implant cut-out

Long nails in this cohort were preferentially used in more complex fracture patterns, particularly AO/OTA 31-A3 fractures, and were associated with longer operative durations and more frequent open reductions. Nail length was nevertheless retained as an exposure variable because it represents a frequent real-world surgical decision in trochanteric fracture care and is relevant for implant standardisation and inventory strategies. Importantly, during the study period long nails were available only in a 130° CCD configuration, whereas short nails were available with multiple CCD options (125°, 130°, 135°), providing a pragmatic setting to explore whether restricted implant configuration compromises technical targets such as TAD and screw placement. Despite this unfavourable case mix, nail length was not significantly associated with achieving TAD < 25 mm or with cut-out risk in multivariable analysis. This absence of effect was confirmed in a predefined homogeneous subgroup of well-reduced AO 31-A2 fractures treated with 130° nails, where nail length again showed no relationship with either endpoint.

These results are consistent with earlier studies reporting that fracture reduction and screw placement outweigh the mechanical influence of nail length [13, 27, 28]. Our findings suggest that nail length per se does not compromise early mechanical outcomes, although residual confounding by indication cannot be fully excluded in this retrospective design. Accordingly, nail length can be selected primarily based on fracture morphology (e.g. distal extension or comminution) and surgeon familiarity without compromising early mechanical outcomes, provided reduction and lag screw placement (center–center with a low tip–apex distance) are optimised.

### CCD angle and anatomical mismatch

In short-nail constructs, we found no independent association between CCD angle (130° vs. non-130°) and either TAD < 25 mm or early cut-out. However, detailed analysis of contralateral anatomy revealed that non-130° implants exhibited greater angular deviation from the patient’s native CCD angle, particularly in short nails with good fracture reduction. This mismatch was associated with a lower probability of achieving TAD < 25 mm, whereas cut-out rates and the proportion of center–center screw positions did not differ significantly between 130° and non-130° implants. Accordingly, mismatch may be better understood as a technical constraint on achieving ideal screw depth/trajectory (and thus TAD) rather than a direct determinant of early cut-out.

These findings suggest that CCD mismatch can make it more challenging to obtain an ideal TAD, especially in anatomies that deviate substantially from the nominal implant angle, but that mismatch does not inevitably translate into early mechanical cut-out if reduction and screw placement are otherwise optimised. Previous work has similarly indicated that mismatch between native CCD angle, femoral anteversion and posterior insertion point of the intramedullary nail may restrict ideal screw routing and increase the technical difficulty of fixation, particularly in patients with low native CCD angles or increased anteversion [20, 29, 30].

Our results are consistent with a nuanced interpretation: within the range of contemporary implants studied, a standard 130° cephalomedullary nail appears adequate for most routine trochanteric fractures when reduction and screw placement are meticulous; however, additional CCD options may still be advantageous in anatomically atypical femora where trajectory constraints make center–center placement with an optimal TAD difficult [20, 31, 32]. Thus, maintaining additional CCD options may be most justifiable in pronounced varus or valgus proximal femora, particularly when achieving a center–center trajectory with an optimal tip–apex distance is not feasible with a standard 130° configuration.

Overall, our data reinforce that procedural success depends primarily on intraoperative technical factors—specifically, accurate reduction, screw trajectory and TAD—rather than on patient phenotype or nail design.

### Strengths and Limitations

This study has several strengths. First, it includes a large, consecutive cohort of predominantly older adults treated at a supra-regional level I trauma center, reflecting real-world practice across a broad spectrum of AO/OTA 31-A1 to 31-A3 fracture patterns. Second, all cases were treated with contemporary cephalomedullary implants, and detailed radiographic assessment was performed using standardised postoperative AP and lateral views, including TAD measurement, Cleveland zone classification, reduction quality and contralateral CCD analysis. Third, the study design incorporated a predefined homogeneous subgroup of well-reduced AO 31-A2 fractures treated with 130° nails to allow biomechanical comparability when evaluating nail length. Finally, postoperative events were captured through systematic review of the institutional electronic record with a predefined 90-day window, and time-to-event analysis was performed to characterise the temporal pattern of early mechanical cut-out. In contrast to many earlier studies that focused solely on crude cut-out rates, our approach integrates detailed CCD mismatch assessment, a homogeneous AO/OTA 31-A2 + 130° subgroup and dedicated 90-day survival analysis, thereby providing a more granular view of how implant geometry and screw positioning interact in the early postoperative period.

The study also has limitations. Its retrospective, single-center design without scheduled outpatient follow-up may have led to under-reporting of cut-outs that occurred within 90 days but were treated exclusively at external institutions. We attempted to mitigate this by delaying data collection until at least six months had elapsed from the last index operation and by reviewing all available inpatient, outpatient, emergency department and radiology records; nevertheless, some events may have been missed. Implant cut-out was defined within 90 days postoperatively, and although no events occurred beyond day 65 in our cohort, late cut-outs beyond 90 days cannot be excluded. The absolute number of cut-out events (*n* = 16) limited statistical power for subgroup and multivariable analyses. Consequently, confidence intervals for estimates of nail length and CCD configuration were wide and the study may have been insufficient to detect smaller effects. Clinically relevant benefit or harm of implant geometry therefore cannot be excluded.

Implant selection, including nail length and CCD angle, was at the discretion of the treating surgeon, introducing potential selection bias. Although we adjusted for fracture type and performed subgroup analyses, residual confounding is likely. Intraoperative details such as entry point modification or subtle changes in screw trajectory were not systematically recorded, and surgeon experience was inferred from team composition rather than individual operator identity [33]. Formal inter- and intraobserver reliability for radiographic measurements was not statistically quantified (e.g., via Intraclass Correlation Coefficient). Although all measurements were duplicated through verification by a senior surgeon to ensure clinical reliability, residual measurement variability cannot be excluded. Because contralateral imaging was not available in all patients (e.g., due to prior contralateral implants or inadequate imaging), the CCD mismatch analyses were restricted to a subset; we did not perform a formal comparison of baseline characteristics between patients with and without eligible contralateral imaging, so selection bias in this subanalysis cannot be excluded. In addition, distal locking configuration and postoperative weight-bearing prescriptions were not available as structured variables and may influence outcomes, potentially contributing to residual confounding [34, 35]. Formal geriatric co-management, infectious complications, and osteoporosis management at discharge were likewise not captured in a standardised manner and could not be analysed. Finally, the generalisability of these findings may be limited to similar high-volume trauma centers with comparable patient demographics and implant availability. Prospective multicenter trials with standardised imaging, longer follow-up and anatomy-based implant planning are needed to confirm these results and to define the subset of patients who may benefit from individualised CCD angle selection.

## Conclusion

Precise center–center lag screw positioning was identified as the primary factor associated with achieving an optimal tip–apex distance (TAD) < 25 mm. Both TAD < 25 mm and center–center screw placement were strongly linked to a reduced risk of early mechanical cut-out and prolonged cut-out-free survival. In contrast, neither femoral nail length nor implant CCD angle showed an independent influence on achieving optimal TAD or on early implant cut-out within the range of contemporary cephalomedullary nails used in this cohort.

Clinically, these findings indicate that meticulous fracture reduction, central screw placement and maintenance of a low TAD represent the most effective intraoperative strategies for minimising mechanical cut-out following cephalomedullary nailing of trochanteric fractures. A standardised 130° cephalomedullary nail appears sufficient for most routine cases, allowing simplification of implant inventories and focusing training efforts on technical execution. However, anatomical mismatch between the native CCD angle and implant angle—particularly in short-nail constructs and in anatomically atypical femora—may reduce the likelihood of achieving an optimal TAD and warrants further investigation. Prospective studies are required to determine whether individualised CCD angle selection or advanced preoperative planning and navigation can further improve clinical and radiological outcomes in complex or atypical cases.

## Supplementary Information

Supplementary Material 1.

Supplementary Material 2.

Supplementary Material 3.

Supplementary Material 4.

Supplementary Material 5.

Supplementary Material 6.

Supplementary Material 7.

Supplementary Material 8.

## Data Availability

The datasets generated and/or analysed during the current study are available from the corresponding author on reasonable request.

## References

[1] Kannus P, Parkkari J, Sievänen H, Heinonen A, Vuori I, Järvinen M. Epidemiology of hip fractures. Bone. 1996;18(1):S57–63. 10.1016/8756-3282(95)00381-9 8717549

[2] Cumming RG, Nevitt MC, Cummings SR. Epidemiology of Hip Fractures. Epidemiol Rev. 1997;19(2):244–57. 9494786 10.1093/oxfordjournals.epirev.a017956

[3] Lohmann R, Frerichmann U, Stöckle U. Proximale Femurfrakturen im Alter. Unfallchirurg. 2007;110(7):603–9. 17503010 10.1007/s00113-007-1257-z

[4] Rapp K, Büchele G, Dreinhöfer K, Bücking B, Becker C, Benzinger P. Epidemiology of hip fractures: Systematic literature review of German data and an overview of the international literature. Z Für Gerontol Geriatr. 2019;52(1):10–6. 10.1007/s00391-018-1382-z PMC6353815 29594444

[5] Bentler SE, Liu L, Obrizan M, Cook EA, Wright KB, Geweke JF, et al. The Aftermath of Hip Fracture: Discharge Placement, Functional Status Change, and Mortality. Am J Epidemiol. 2009;170(10):1290–9. 19808632 10.1093/aje/kwp266 PMC2781759

[6] Wolinsky FD, Fitzgerald JF, Stump TE. The effect of hip fracture on mortality, hospitalization, and functional status: a prospective study. Am J Public Health. 1997;87(3):398–403. 9096540 10.2105/ajph.87.3.398 PMC1381011

[7] Pincus D, Ravi B, Wasserstein D, Huang A, Paterson JM, Nathens AB, et al. Association Between Wait Time and 30-Day Mortality in Adults Undergoing Hip Fracture Surgery. JAMA. 2017;318(20):1994. 29183076 10.1001/jama.2017.17606 PMC5820694

[8] Simunovic N, Devereaux PJ, Sprague S, Guyatt GH, Schemitsch E, DeBeer J, et al. Effect of early surgery after hip fracture on mortality and complications: systematic review and meta-analysis. Can Med Assoc J. 2010;182(15):1609–16. 20837683 10.1503/cmaj.092220 PMC2952007

[9] Forte ML, Virnig BA, Kane RL, Durham S, Bhandari M, Feldman R, et al. Geographic Variation in Device Use for Intertrochanteric Hip Fractures. J Bone Jt Surg-Am Vol. 2008;90(4):691–9. 10.2106/JBJS.G.00414 18381304

[10] Sandmann GH, Biberthaler P. Pertrochantäre Femurfrakturen beim geriatrischen Patienten. Unfallchirurg. 2015;118(5):447–62. 25964023 10.1007/s00113-015-0007-x

[11] Swift B, Stewart A, Grammatopoulos G, Papp S, Wilkin G, Liew A. Comparing the rates and modes of failure of two third generation cephalomedullary nail systems in the treatment of intertrochanteric hip fractures. Injury. 2022;53(8):2846–52. 35725507 10.1016/j.injury.2022.06.005

[12] Han SB, Jung JK, Jang CY, Kwak DK, Kim JW, Yoo JH. Gamma3 nail with U-Blade (RC) lag screw is effective with better surgical outcomes in trochanteric hip fractures. Sci Rep. 2020;10(1):6021. 32265481 10.1038/s41598-020-62980-2 PMC7138836

[13] De Bruijn K, Den Hartog D, Tuinebreijer W, Roukema G. Reliability of Predictors for Screw Cutout in Intertrochanteric Hip Fractures. J Bone Jt Surg. 2012;94(14):1266–72. 10.2106/JBJS.K.00357 22810396

[14] Baumgaertner MR, Curtin SL, Keggi JM. The value of the tip–apex distance in predicting failure of fixation of peritrochanteric fractures of the hip. J Bone Jt Surg Am. 1995;77(7):1058–64. 10.2106/00004623-199507000-00012 7608228

[15] Rubio-Avila J, Madden K, Simunovic N, Bhandari M. Tip to apex distance in femoral intertrochanteric fractures: a systematic review. J Orthop Sci. 2013;18(4):592–8. 23636573 10.1007/s00776-013-0402-5

[16] Caruso G, Corradi N, Caldaria A, Bottin D, Lo Re D, Lorusso V, et al. New tip-apex distance and calcar-referenced tip-apex distance cut-offs may be the best predictors for cut-out risk after intramedullary fixation of proximal femur fractures. Sci Rep. 2022;12(1):357. 35013492 10.1038/s41598-021-04252-1 PMC8748913

[17] Yam M, Chawla A, Kwek E. Rewriting the tip apex distance for the proximal femoral nail anti-rotation. Injury. 2017;48(8):1843–7. 28689807 10.1016/j.injury.2017.06.020

[18] Stern LC, Gorczyca JT, Kates S, Ketz J, Soles G, Humphrey CA. Radiographic Review of Helical Blade Versus Lag Screw Fixation for Cephalomedullary Nailing of Low-Energy Peritrochanteric Femur Fractures: There is a Difference in Cutout. J Orthop Trauma. 2017;31(6):305–10. 28346314 10.1097/BOT.0000000000000853

[19] Rogers MJ, King TL, Kim J, Adeyemi TF, Higgins TF, Maak TG. Femoral Neck Shaft Angle and Management of Proximal Femur Fractures: Is the Contralateral Femur a Reliable Template? J Orthop Trauma. 2021;35(10):529–34. 33813545 10.1097/BOT.0000000000002069 PMC10506416

[20] Parry JA, Barrett I, Schoch B, Yuan B, Cass J, Cross W. Does the Angle of the Nail Matter for Pertrochanteric Fracture Reduction? Matching Nail Angle and Native Neck-Shaft Angle. J Orthop Trauma. 2018;32(4):174–7. 29377850 10.1097/BOT.0000000000001096

[21] Cleveland M, Bosworth DM, Thompson FR, Wilson HJ Jr, Ishizuka T. A ten-year analysis of intertrochanteric fractures of the femur. Clevel M Bosworth DM Thompson FR Wilson HJ Jr Ishizuka T. J Bone Joint Surg Am. 1959;41-A(8):1399–408. 13849408

[22] Cuschieri S. The STROBE guidelines. Saudi J Anaesth. 2019;13(5):31. 10.4103/sja.SJA_543_18 PMC6398292 30930717

[23] Mavrogenis AF, Panagopoulos GN, Megaloikonomos PD, Igoumenou VG, Galanopoulos I, Vottis CT et al. Complications After Hip Nailing for Fractures. Orthopedics [Internet]. 2016 Jan [cited 2026 Feb 15];39(1). Available from: https://journals.healio.com/doi/10.3928/01477447-20151222-11 10.3928/01477447-20151222-11 26726984

[24] Ciufo DJ, Zaruta DA, Lipof JS, Judd KT, Gorczyca JT, Ketz JP. Risk Factors Associated With Cephalomedullary Nail Cutout in the Treatment of Trochanteric Hip Fractures. J Orthop Trauma. 2017;31(11):583–8. 28827502 10.1097/BOT.0000000000000961

[25] Caruso G, Bonomo M, Valpiani G, Salvatori G, Gildone A, Lorusso V, et al. A six-year retrospective analysis of cut-out risk predictors in cephalomedullary nailing for pertrochanteric fractures: Can the tip-apex distance (TAD) still be considered the best parameter? Bone Jt Res. 2017;6(8):481–8. 10.1302/2046-3758.68.BJR-2016-0299.R1 PMC5579311 28790037

[26] Kuzyk PRT, Zdero R, Shah S, Olsen M, Waddell JP, Schemitsch EH. Femoral Head Lag Screw Position for Cephalomedullary Nails: A Biomechanical Analysis. J Orthop Trauma. 2012;26(7):414–21. 22337483 10.1097/BOT.0b013e318229acca

[27] Shannon SF, Yuan BJ, Cross WW, Barlow JD, Torchia ME, Holte PK, et al. Short Versus Long Cephalomedullary Nails for Pertrochanteric Hip Fractures: A Randomized Prospective Study. J Orthop Trauma. 2019;33(10):480–6. 31232891 10.1097/BOT.0000000000001553

[28] Dunn J, Kusnezov N, Bader J, Waterman BR, Orr J, Belmont PJ. Long versus short cephalomedullary nail for trochanteric femur fractures (OTA 31-A1, A2 and A3): a systematic review. J Orthop Traumatol. 2016;17(4):361–7. 27093971 10.1007/s10195-016-0405-z PMC5071234

[29] Yoshitani J, Kabata T, Kajino Y, Inoue D, Ohmori T, Ueoka K, et al. Correlation between lag screw route and the ideal insertion point of the intramedullary nail. Sci Rep. 2021;11(1):13750. 34215831 10.1038/s41598-021-93348-9 PMC8253735

[30] Jing Y, Chang L, Cong B, Wang J, Chen M, Tang Z, et al. Preoperative 3D printing planning technology combined with orthopedic surgical robot-assisted minimally invasive screw fixation for the treatment of pelvic fractures: a retrospective study. PeerJ. 2024;12:e18632. 39677955 10.7717/peerj.18632 PMC11646416

[31] Fisher ND, Parola R, Anil U, Herbosa C, Boadi B, Ganta A, et al. A Good Tip-Apex Distance Does Not Make Up For a Poor Reduction in Intertrochanteric Hip Fractures Treated with an Cephalomedullary Nail: The Utility of the Neck-Shaft Angle in Preventing Fixation Failure. J Am Acad Orthop Surg. 2024;32(2):83–91. 37748038 10.5435/JAAOS-D-22-00972

[32] Yoon YC, Lee SJ, Song HK. Biomechanical Analysis of Fixation Strength in Unstable Intertrochanteric Femoral Fracture Models Based on the Caput–Collum–Diaphyseal Angle of Cephalomedullary Nails and Position of Lag Screws. J Clin Med. 2025;14(18):6495. 41010700 10.3390/jcm14186495 PMC12470287

[33] Robles AS, Najdawi JJ, Wang J, Rockov ZA, Parikh HB, Little MTM, et al. Optimizing the Entry Point for Reconstruction Nailing of the Femur. J Am Acad Orthop Surg. 2023;31(18):e721–6. 37205875 10.5435/JAAOS-D-22-00778

[34] Hernández-Pascual C, Santos-Sánchez JÁ, García-González JM, Silva-Viamonte CF, Pablos-Hernández C, Ramos-Pascua L, et al. Long-term outcomes of distal locking in extracapsular fractures treated with trochanteric Gamma3 nails. J Orthop Traumatol. 2021;22(1):48. 34825977 10.1186/s10195-021-00609-4 PMC8620307

[35] Hernández-Pascual C, Santos-Sánchez JÁ, Hernández-Rodríguez J, Silva-Viamonte CF, Pablos-Hernández C, Alonso-Rodríguez P, et al. Partial weight bearing and long-term survival outcomes in extracapsular hip fractures treated with trochanteric Gamma3 nails. BMC Musculoskelet Disord. 2025;26(1):129. 39920603 10.1186/s12891-024-08043-3 PMC11804022

