# Supplementary material for: Influence of cephalomedullary nail length and caput–collum–diaphyseal angle on tip–apex distance and early mechanical cut-out in trochanteric femur fractures
Source: BMC Musculoskelet Disord. 2026 Mar 7;27:290. doi: 10.1186/s12891-026-09685-1 (PMC13063900; doi:10.1186/s12891-026-09685-1)
Supplement: Supplementary file 1 — Supplementary Material 1. [file 12891_2026_9685_MOESM1_ESM.docx]

**Supplementary Table S3. Intraoperative complication categories by nail length**

| **Complication category** | **Long nails (n=124)** | **Short nails (n=249)** | **P-value** |
| --- | --- | --- | --- |
| Any intraoperative complication | 30 (24.2%) | 28 (11.2%) | 0.002 |
| Technical/surgical problem | 20 (16.1%) | 13 (5.2%) | <0.001 |
| Instrumentation/implantation problem | 3 (2.4%) | 2 (0.8%) | 0.338 |
| Hemodynamic instability | 3 (2.4%) | 4 (1.6%) | 0.690 |
| Coagulation-related event | 2 (1.6%) | 2 (0.8%) | 0.603 |
| Other intraoperative complication | 2 (1.6%) | 3 (1.2%) | 1.000 |
| Operative delay | 0 (0.0%) | 4 (1.6%) | 0.306 |

Values are n (%). P-values are from two-sided Fisher’s exact tests comparing long vs short nails for each row. Categories were derived from operative-report documentation and are not necessarily mutually exclusive.
