# Supplementary material for: Influence of cephalomedullary nail length and caput–collum–diaphyseal angle on tip–apex distance and early mechanical cut-out in trochanteric femur fractures
Source: BMC Musculoskelet Disord. 2026 Mar 7;27:290. doi: 10.1186/s12891-026-09685-1 (PMC13063900; doi:10.1186/s12891-026-09685-1)
Supplement: Supplementary file 2 — Supplementary Material 2. [file 12891_2026_9685_MOESM2_ESM.docx]

**Supplementary Table S1. Baseline comorbidity categories by nail length**

| **Comorbidity category** | **Long nails (n=124) None / Single / Multiple** | **Short nails (n=249) None / Single / Multiple** | **P-value** |
| --- | --- | --- | --- |
| Vascular comorbidity | 98 (79.0%) / 17 (13.7%) / 9 (7.3%) | 175 (70.3%) / 30 (12.0%) / 44 (17.7%) | 0.025 |
| Pulmonary comorbidity | 105 (84.7%) / 15 (12.1%) / 4 (3.2%) | 215 (86.3%) / 29 (11.6%) / 5 (2.0%) | 0.760 |
| Gastroenterological comorbidity | 102 (82.3%) / 16 (12.9%) / 6 (4.8%) | 211 (84.7%) / 25 (10.0%) / 13 (5.2%) | 0.704 |
| Hepatic comorbidity | 118 (95.2%) / 5 (4.0%) / 1 (0.8%) | 238 (95.6%) / 10 (4.0%) / 1 (0.4%) | 0.880 |
| Renal comorbidity | 96 (77.4%) / 27 (21.8%) / 1 (0.8%) | 168 (67.5%) / 75 (30.1%) / 6 (2.4%) | 0.111 |
| Thyroid disease | 111 (89.5%) / 13 (10.5%) / 0 (0.0%) | 208 (83.5%) / 41 (16.5%) / 0 (0.0%) | 0.164 |
| Cerebral disease | 121 (97.6%) / 2 (1.6%) / 1 (0.8%) | 247 (99.2%) / 2 (0.8%) / 0 (0.0%) | 0.850 |
| Spinal pathologies | 117 (94.4%) / 7 (5.6%) / 0 (0.0%) | 246 (98.8%) / 3 (1.2%) / 0 (0.0%) | 0.031 |
| Autoimmune disease | 122 (98.4%) / 2 (1.6%) / 0 (0.0%) | 249 (100.0%) / 0 (0.0%) / 0 (0.0%) | 1.000 |
| Metabolic disease | 71 (57.3%) / 35 (28.2%) / 18 (14.5%) | 124 (49.8%) / 92 (36.9%) / 33 (13.3%) | 0.243 |
| Malignant disease | 104 (83.9%) / 19 (15.3%) / 1 (0.8%) | 203 (81.5%) / 41 (16.5%) / 5 (2.0%) | 0.649 |
| Psychiatric comorbidity | 108 (87.1%) / 11 (8.9%) / 5 (4.0%) | 222 (89.2%) / 21 (8.4%) / 6 (2.4%) | 0.671 |
| Neurological comorbidity | 84 (67.7%) / 27 (21.8%) / 13 (10.5%) | 152 (61.0%) / 62 (24.9%) / 35 (14.1%) | 0.418 |
| ENT disease | 122 (98.4%) / 1 (0.8%) / 1 (0.8%) | 233 (93.6%) / 15 (6.0%) / 1 (0.4%) | 0.058 |
| Eye disease | 116 (93.5%) / 7 (5.6%) / 1 (0.8%) | 226 (90.8%) / 19 (7.6%) / 4 (1.6%) | 0.627 |
| Extremity pathologies | 68 (54.8%) / 29 (23.4%) / 27 (21.8%) | 155 (62.2%) / 49 (19.7%) / 45 (18.1%) | 0.388 |
| Hematological disease | 123 (99.2%) / 1 (0.8%) / 0 (0.0%) | 234 (94.0%) / 14 (5.6%) / 1 (0.4%) | 0.064 |

Values are n (%). Each comorbidity category was coded as none / single / multiple diagnoses. P-values as originally computed (Chi-square tests).
