# Supplementary material for: Influence of cephalomedullary nail length and caput–collum–diaphyseal angle on tip–apex distance and early mechanical cut-out in trochanteric femur fractures
Source: BMC Musculoskelet Disord. 2026 Mar 7;27:290. doi: 10.1186/s12891-026-09685-1 (PMC13063900; doi:10.1186/s12891-026-09685-1)
Supplement: Supplementary file 3 — Supplementary Material 3. [file 12891_2026_9685_MOESM3_ESM.docx]

**Supplementary Table S2. Medication categories at admission by nail length**

| **Medication category** | **Long nails (n=124)** | **Short nails (n=249)** | **P-value** |
| --- | --- | --- | --- |
| Any medication at admission (OAK and/or osteoporosis therapy) | 75 (60.5%) | 167 (67.1%) | 0.254 |
| Oral anticoagulation (OAK) | 62 (50.0%) | 140 (56.2%) | 0.305 |
| Osteoporosis medication | 33 (26.6%) | 78 (31.3%) | 0.414 |
| Combined OAK + osteoporosis medication | 20 (16.1%) | 51 (20.5%) | 0.385 |

Values are n (%). P-values as originally computed (Chi-square tests).
