# Supplementary material for: Influence of cephalomedullary nail length and caput–collum–diaphyseal angle on tip–apex distance and early mechanical cut-out in trochanteric femur fractures
Source: BMC Musculoskelet Disord. 2026 Mar 7;27:290. doi: 10.1186/s12891-026-09685-1 (PMC13063900; doi:10.1186/s12891-026-09685-1)
Supplement: Supplementary file 4 — Supplementary Material 4. [file 12891_2026_9685_MOESM4_ESM.docx]

**Supplementary Table S4. Postoperative medical complications by nail length**

| **Medical complication** | **Long nails (n=124)** | **Short nails (n=249)** | **P-value** |
| --- | --- | --- | --- |
| Anemia | 25 (20.2%) | 43 (17.3%) | 0.590 |
| Urinary tract infection (UTI) | 10 (8.1%) | 47 (18.9%) | 0.010 |
| Pneumonia | 6 (4.8%) | 11 (4.4%) | 1.000 |
| Gastrointestinal complications | 5 (4.0%) | 10 (4.0%) | 1.000 |
| Clostridium enteritis | 2 (1.6%) | 2 (0.8%) | 0.856 |
| Cardiac complications | 7 (5.6%) | 9 (3.6%) | 0.522 |
| Respiratory complications | 3 (2.4%) | 6 (2.4%) | 1.000 |
| Renal complications | 10 (8.1%) | 17 (6.8%) | 0.824 |
| Electrolyte disorder | 5 (4.0%) | 15 (6.0%) | 0.575 |
| Neurological complications | 4 (3.2%) | 13 (5.2%) | 0.544 |
| Thromboembolic events | 1 (0.8%) | 2 (0.8%) | 1.000 |
| Hepatic complications | 2 (1.6%) | 2 (0.8%) | 0.856 |

Values are n (%). P-values as originally computed (Chi-square tests).
