# Supplementary material for: Influence of cephalomedullary nail length and caput–collum–diaphyseal angle on tip–apex distance and early mechanical cut-out in trochanteric femur fractures
Source: BMC Musculoskelet Disord. 2026 Mar 7;27:290. doi: 10.1186/s12891-026-09685-1 (PMC13063900; doi:10.1186/s12891-026-09685-1)
Supplement: Supplementary file 5 — Supplementary Material 5. [file 12891_2026_9685_MOESM5_ESM.docx]

**Supplementary Fig. S1** Multivariable logistic regression predicting TAD < 25 mm (n = 373). Forest plot illustrating odds ratios (OR) with 95% confidence intervals (CI) from a multivariable logistic regression model evaluating predictors of achieving a TAD < 25 mm. The dashed vertical line represents an OR of 1. Error bars indicate 95% CI. Statistically significant predictors are marked with asterisks. ¹ Nail length: short vs. long nail; ² Screw position: center vs. non-center, according to modified Cleveland zones [21]; ³ Age: per year increase; ⁴ Fracture classification: AO/OTA A3 vs. A1/A2; ⁵ Reduction quality: poor vs. good, according to Baumgaertner et al. [14]; * p < 0.05, ** p < 0.01, *** p < 0.001

**Supplementary Fig. S2** Multivariable logistic regression predicting TAD < 25 mm in short nails (n = 249). Forest plot of predictors for achieving a TAD < 25 mm in patients treated with short cephalomedullary nails. Odds ratios (OR) and 95% confidence intervals (CI) were calculated using multivariable logistic regression. The dashed vertical line indicates an OR of 1. ¹ CCD angle: 130° vs. non-130° implant CCD angle; ² Screw position: center vs. non-center, according to modified Cleveland zones [21]; ³ Reduction quality: poor vs. good, according to Baumgaertner et al. [14]; * p < 0.05, ** p < 0.01, *** p < 0.001

**Supplementary Fig. S3** Firth-penalized logistic regression predicting implant cut-out (≤ 90 days). Forest plot generated from a Firth-penalized logistic regression model showing predictors of implant cut-out occurring within 90 days postoperatively. Where appropriate, estimates are reported separately for all nail types and for short nails. Odds ratios (OR) with 95% confidence intervals (CI) are displayed on a logarithmic scale. ¹ TAD (all nails): < 25 mm vs. ≥ 25 mm; ² TAD (short nails): < 25 mm vs. ≥ 25 mm; ³ Screw position (all nails): center vs. non-center, according to modified Cleveland zones [21]; ⁴ Screw position (short nails): center vs. non-center, according to modified Cleveland zones [21]; ⁵ Nail length: short vs. long nail; ⁶ CCD angle: 130° vs. non-130° implant CCD angle (short nails only); * p < 0.05, ** p < 0.01, *** p < 0.001
