# Supplementary figures and images for: Influence of cephalomedullary nail length and caput–collum–diaphyseal angle on tip–apex distance and early mechanical cut-out in trochanteric femur fractures
Source: BMC Musculoskelet Disord. 2026 Mar 7;27:290. doi: 10.1186/s12891-026-09685-1 (PMC13063900; doi:10.1186/s12891-026-09685-1)

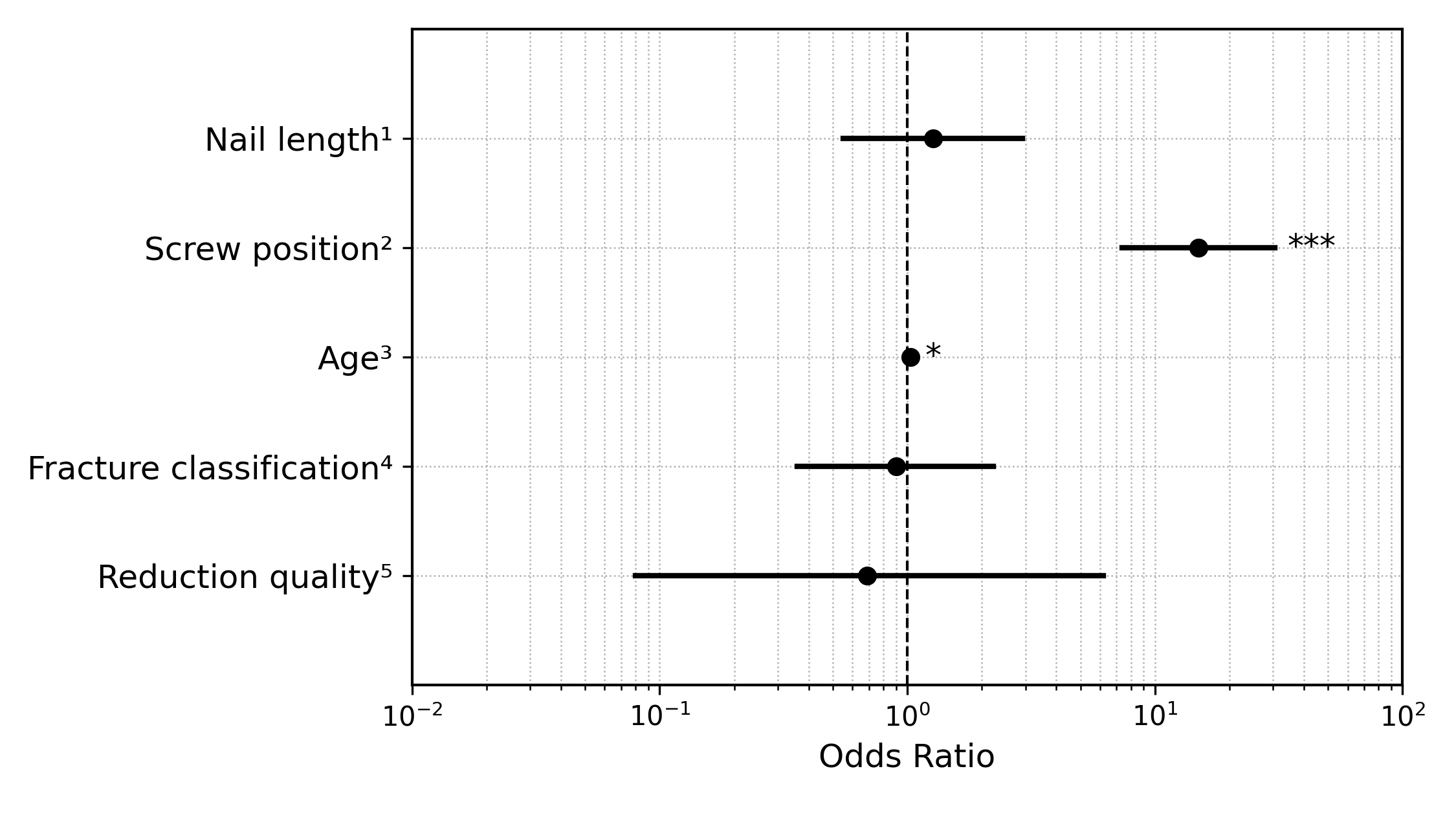

Supplement: Supplementary file 6 — Supplementary Material 6. [file 12891_2026_9685_MOESM6_ESM.tiff]

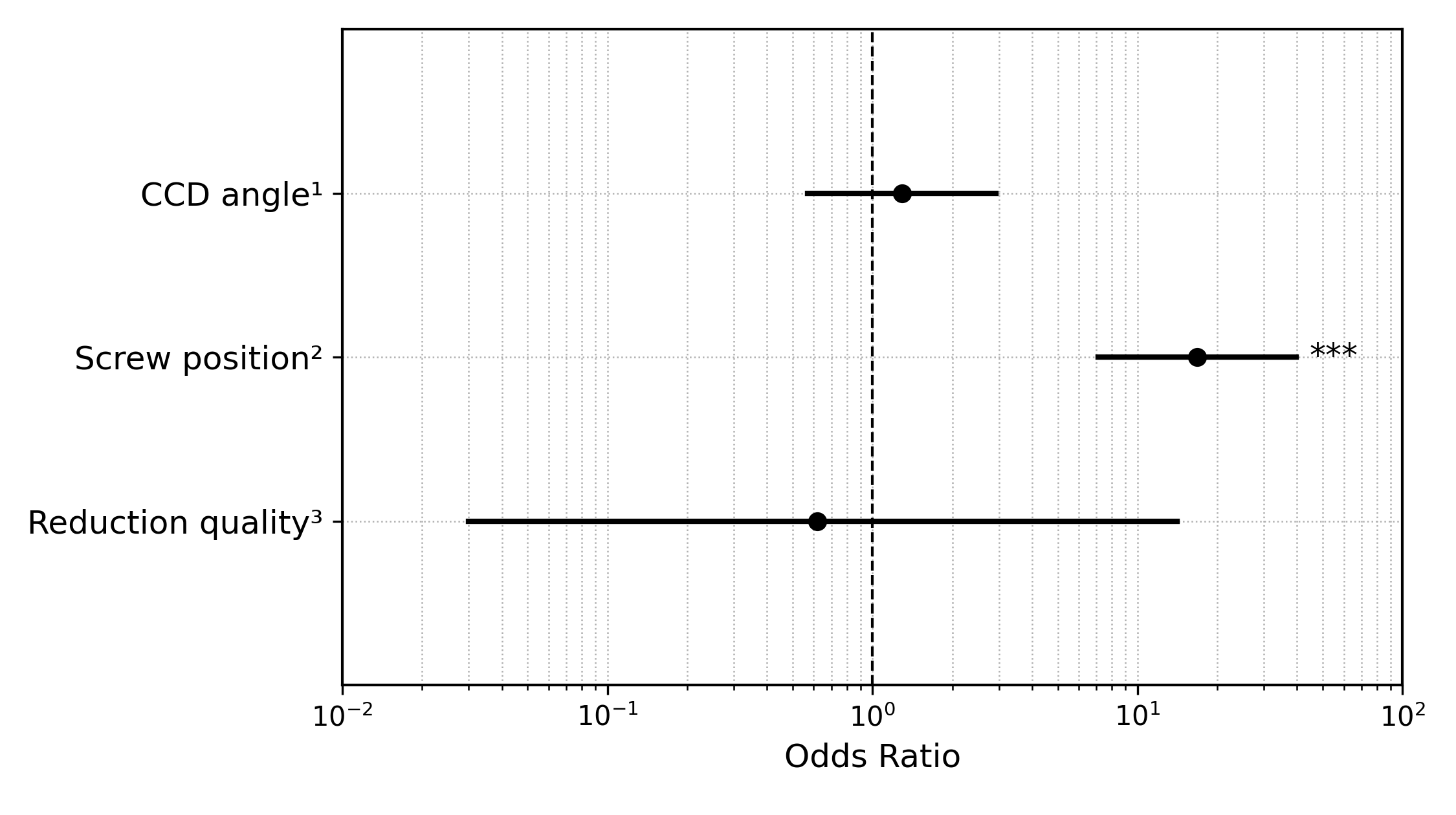

Supplement: Supplementary file 7 — Supplementary Material 7. [file 12891_2026_9685_MOESM7_ESM.tiff]

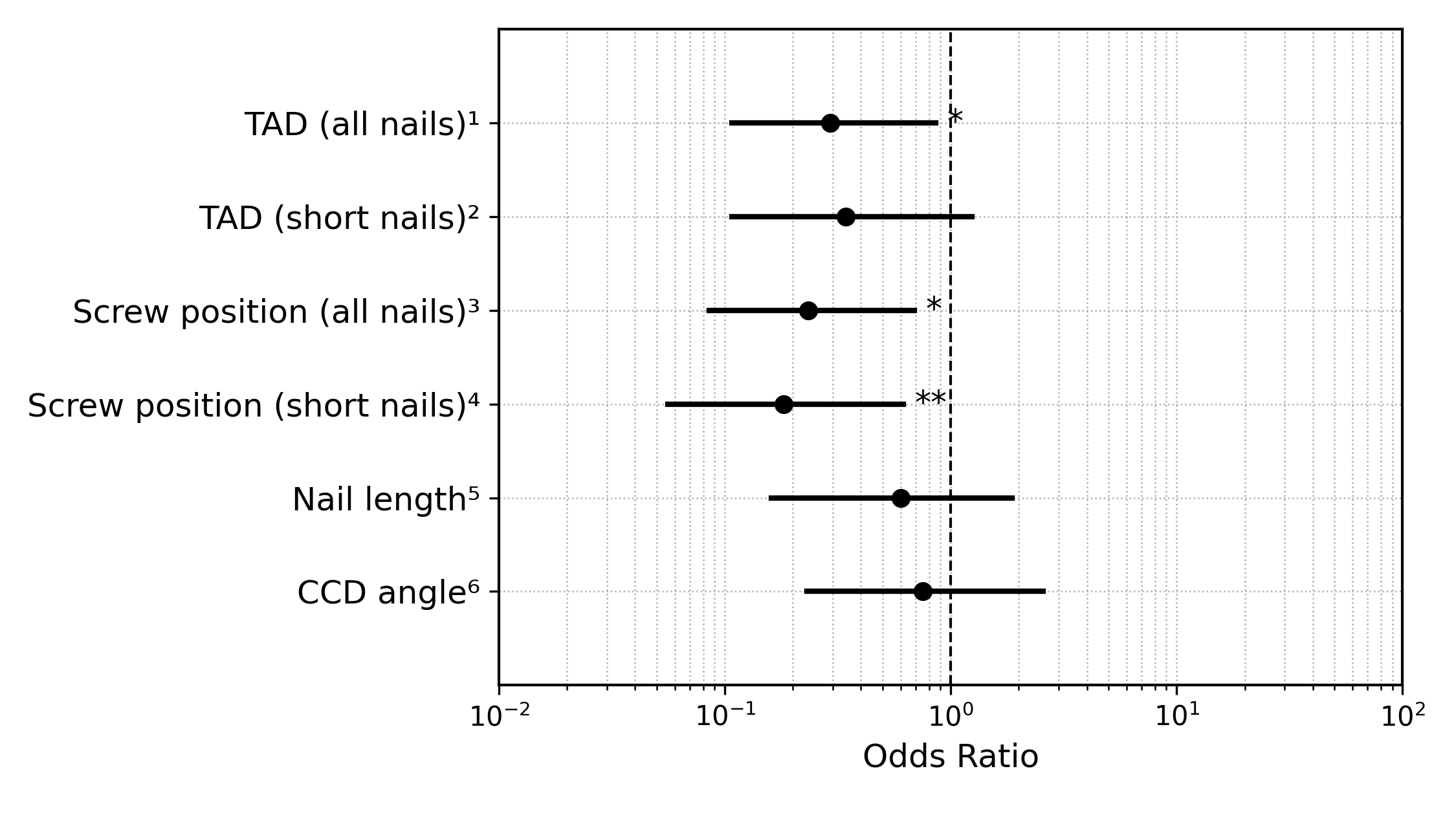

Supplement: Supplementary file 8 — Supplementary Material 8. [file 12891_2026_9685_MOESM8_ESM.tiff]
